# Maternal and infant renal safety following tenofovir disoproxil fumarate exposure during pregnancy in a randomized control trial

**DOI:** 10.1186/s12879-022-07608-8

**Published:** 2022-07-20

**Authors:** Kristin Baltrusaitis, Bonus Makanani, Camlin Tierney, Mary Glenn Fowler, Dhayendre Moodley, Gerhard Theron, Lynette H. Nyakudya, Musunga Tomu, Lee Fairlie, Kathleen George, Barbara Heckman, Kevin Knowles, Renee Browning, George K. Siberry, Taha E. Taha, Lynda Stranix-Chibanda, for the PROMISE P1084s Study Team, Lynda Stranix-Chibanda, Judith Currier, Katherine Luzuriaga, Adriana Weinberg, James McIntyre, Tsungai Chipato, Karin Klingman, Renee Browning, Mireille Mpoudi-Ngole, Jennifer S. Read, George Siberry, Heather Watts, Lynette Purdue, Terrence Fenton, Linda Barlow-Mosha, Mary Pat Toye, Mark Mirochnick, William B. Kabat, Benjamin Chi, Marc Lallemant, Karin Nielsen, Kevin Butler, Konstantia Angelidou, David Shapiro, Sean Brummel, Anne Coletti, Veronica Toone, Megan Valentine, Kathleen George, Amanda Zadzilka, Michael Basar, Amy Jennings, Adam Manzella, Sandesh Patil, Ramesh Bhosale, Neetal Nevreka, Salome Kunje, Alex Siyasiya, Mervis Maulidi, Francis Martinson, Ezylia Makina, Beteniko Milala, Nozibusiso Rejoice Skosana, Sajeeda Mawlana, Jeanne Louw, Magdel Rossouw, Lindie Rossouw, Masebole Masenya, Janet Grab, Nasreen Abrahams, Mandisa Nyati, Sylvia Dittmer, Dhayendre Moodley, Vani Chetty, Alicia Catherine Desmond, Boniface Njau, Cynthia Asiyo, Pendo Mlay, Maxensia Owor, Moreen Kamateeka, Dorothy Sebikari, Tichaona Vhembo, Nyasha Mufukari, Lynda Stranix-Chibanda, Teacler Nematadzira, Gift Chareka, Jean Dimairo, Tsungai Chipato, Bangani Kusakara, Mercy Mutambanengwe, Emmie Marote

**Affiliations:** 1 Center for Biostatistics in AIDS Research, Harvard T.H. Chan School of Public Health, 651 Huntington Avenue, Boston, MA 02115 USA; 2 College of Medicine, University of Malawi, Blantyre, Malawi; 3 Department of Pathology, Johns Hopkins University, Baltimore, MD USA; 4 Centre for AIDS Prevention Research in South Africa and Department of Obstetrics and Gynecology, School of Clinical Medicine, University of KwaZulu Natal, Durban, South Africa; 5 Department of Obstetrics and Gynaecology, Faculty of Medicine and Health Sciences, Stellenbosch University, Cape Town, South Africa; 6 University of Zimbabwe Clinical Trials Research Centre, Harare, Zimbabwe; 7 Wits RHI, Faculty of Health Sciences, University of the Witwatersrand, Johannesburg, South Africa; 8 FHI 360, Durham, NC USA; 9 Frontier Science Foundation, Amherst, NY USA; 10 National Institute of Allergy and Infectious Diseases, National Institutes of Health, Bethesda, MD USA; 11 United States Agency for International Development, Washington, DC USA; 12 Department of Epidemiology, Johns Hopkins Bloomberg School of Public Health, Baltimore, MD USA; 13 Child and Adolescent Health Unit, Faculty of Medicine and Health Sciences, University of Zimbabwe, Harare, Zimbabwe; 14 Division of Infectious Diseases, David Geffen School of Medicine, University of California, Los Angeles, CA USA; 15 Program in Molecular Medicine, University of Massachusetts Medical School Worcester, Worcester, MA USA; 16 Pediatrics-Infectious Diseases, School of Medicine, University of Colorado, Children’s Hospital Colorado, Aurora, CO USA; 17 Anova Health Institute, Johannesburg, South Africa; 18 Division of Epidemiology and Biostatistics, School of Public Health and Family Medicine, University of Cape Town, Cape Town, South Africa; 19 Department of Obstetrics and Gynecology, University of Zimbabwe, Harare, Zimbabwe; 20 Division of AIDS, National Institute of Allergy and Infectious Diseases, National Institutes of Health, Bethesda, MD USA; 21 Department of Epidemiology and Biostatistics, University of California at San Francisco, San Francisco, CA USA; 22 Office of the Global AIDS Coordinator, Department of State, Washington, DC USA; 23 Makerere University-Johns Hopkins University Research Collaboration CRS, Kampala, Uganda; 24 Division of Neonatology, School of Medicine, Boston University, Boston, MA USA; 25 Ann and Robert H. Lurie Children’s Hospital of Chicago, Chicago, IL USA; 26 University of North Carolina at Chapel Hill, Chapel Hill, NC USA; 27 AMS-PHPT Research Collaboration, Faculty of Associated Medical Sciences, Chiang Mai University, Chiang Mai, Thailand; 28 Harvard School of Public Health, Boston, MA USA; 29 Institut de Recherché Pour Le Développement (IRD), UMI 174-PHPT, Marseille, France; 30 Department of Pediatrics, David Geffen UCLA School of Medicine, Los Angeles, CA USA; 31 Byramjee Jeejeebhoy Medical College, Pune, Maharashtra India; 32 College of Medicine JHU CRS, Queen Elizabeth Central Hospital, Blantyre, Malawi; 33 University of North Carolina Lilongwe, Kamuzu Central Hospital/Tidziwe Centre, Lilongwe, Malawi; 34 Nelson R. Mandela School of Medicine, University of KwaZulu-Natal, Durban, South Africa; 35 Stellenbosch University KIDCRU, Cape Town, South Africa; 36 Wits RHI Shandukani Research Centre, Harriet Shezi Children’s Clinic, Johannesburg, South Africa; 37 Soweto IMPAACT CRS, Chris Hani Baragwanath Hospital, Johannesburg, South Africa; 38 Umlazi Clinical Research Site, KwaZulu-Natal, South Africa; 39 Kilimanjaro Christian Medical Center, Moshi, Tanzania; 40 MU-JHU Care Limited, Kampala, Uganda; 41 Harare Family Care, Harare, Zimbabwe; 42 St Mary’s Clinic, Chitungwiza, Zimbabwe

**Keywords:** HIV/AIDS, Antiretroviral therapy, Renal function, Pregnancy, Prevention of perinatal HIV transmission

## Abstract

**Background:**

Tenofovir disoproxil fumarate (TDF) in combination with other antiretroviral (ARV) drugs has been in clinical use for HIV treatment since its approval in 2001. Although the effectiveness of TDF in preventing perinatal HIV infection is well established, information about renal safety during pregnancy is still limited.

**Trial design:**

The IMPAACT PROMISE study was an open-label, strategy trial that randomized pregnant women to one of three arms: TDF based antiretroviral therapy (ART), zidovudine (ZDV) based ART, and ZDV alone (standard of care at start of enrollment). The P1084s substudy was a nested, comparative study of renal outcomes in women and their infants.

**Methods:**

PROMISE participants (n = 3543) were assessed for renal dysfunction using calculated creatinine clearance (CrCl) at study entry (> 14 weeks gestation), delivery, and postpartum weeks 6, 26, and 74. Of these women, 479 were enrolled in the P1084s substudy that also assessed maternal calcium and phosphate as well as infant calculated CrCl, calcium, and phosphate at birth.

**Results:**

Among the 1338 women who could be randomized to TDF, less than 1% had a baseline calculated CrCl below 80 mL/min. The mean (standard deviation) maternal calculated CrCl at delivery in the TDF-ART arm [147.0 mL/min (51.4)] was lower than the ZDV-ART [155.0 mL/min (43.3); primary comparison] and the ZDV Alone [158.5 mL/min (45.0)] arms; the mean differences (95% confidence interval) were − 8.0 mL/min (− 14.5, − 1.5) and − 11.5 mL/min (− 18.0, − 4.9), respectively. The TDF-ART arm had lower mean maternal phosphate at delivery compared with the ZDV-ART [− 0.14 mg/dL (− 0.28, − 0.01)] and the ZDV Alone [− 0.17 mg/dL (− 0.31, − 0.02)] arms, and a greater percentage of maternal hypophosphatemia at delivery (4.23%) compared with the ZDV-ART (1.38%) and the ZDV Alone (1.46%) arms. Maternal calcium was similar between arms. In infants, mean calculated CrCl, calcium, and phosphate at birth were similar between arms (all CIs included 0).

**Conclusions:**

Although mean maternal calculated CrCl at Delivery was lower in the TDF-ART arm, the difference between arms is unlikely to be clinically significant. During pregnancy, the TDF-ART regimen had no observed safety concerns for maternal or infant renal function.

*Trial Registration*: NCT01061151 on 10/02/2010 for PROMISE (1077BF). NCT01066858 on 10/02/2010 for P1084s.

**Supplementary Information:**

The online version contains supplementary material available at 10.1186/s12879-022-07608-8.

## Background

Since its approval by the US Food and Drug Administration (FDA) in 2001, tenofovir disoproxil fumarate (TDF) has been in clinical use as treatment for HIV-1 infection in combination with other antiretroviral (ARV) drugs, pre-exposure prophylaxis (PrEP) for prevention of sexually acquired HIV-1 infection, and treatment of chronic hepatitis B virus (HBV) infection [1, 2]. Because TDF has a favorable potency, tolerability, and pharmacokinetic profile that allows for daily dosing, it is one of the most commonly used ARVs in adolescents and adults [3].

The safety data on TDF is primarily from adult randomized treatment trials and clinical experience [4–14]. The main toxicities include bone demineralization and renal toxicity [3]. Cases of nephrotoxicity (i.e., Fanconi syndrome including hypophosphatemia, renal insufficiency, acute tubular necrosis, and acute renal failure) have been reported in adults receiving TDF [11–16]. Of note, tubular dysfunction in the absence of decline in estimated glomerular filtration rate (eGFR) appears to occur more frequently than frank renal insufficiency [17]. Renal toxicity attributed to TDF has also been reported in young children treated with TDF and in TDF PrEP studies, and TDF has been associated with renal dysfunction in United States (US) and United Kingdom (UK)-based pediatric cohort studies [18, 19]. Although review of changes in renal parameters in over 1,000 adults in randomized trials revealed small decrements in eGFR in TDF patients compared with non-TDF patients over 3 years, clinically significant TDF-associated renal toxicity is rarely observed in adults [20].

TDF ARV therapy is also the recommended strategy to prevent vertical transmission of HIV and HBV as well as for HIV prevention, including for PrEP, in pregnant and breastfeeding women [21–23]. In the UK, a retrospective cohort analysis of 71 pregnant women receiving TDF showed no decline in renal function during pregnancy and normal renal function (eGFR > 90 mL/min) at 6 weeks postpartum, except for one woman who had a postpartum eGFR of 60 mL/min [24]. However, renal insufficiency and Fanconi syndrome associated with maternal TDF use have been reported in perinatally infected children [25, 26].

For many women, TDF is an effective and well-tolerated part of a combination ARV regimen that treats maternal illness (HIV, HBV, or both) and prevents vertical transmission antepartum, perinatally, and through breast milk. However, because the renal effects of prolonged maternal TDF use in pregnant women and their infants is limited, additional safety data are needed to inform clinical use. This analysis compares renal outcome measures in a randomized clinical trial of TDF and non-TDF-containing treatment regimens during pregnancy for pregnant women living with HIV with high CD4 counts and their infants.

## Methods

### Trial design

This analysis is a randomized comparison of the effects of maternal TDF or no maternal TDF during pregnancy on the renal health of women who participated in the Antepartum (AP) Component of the IMPAACT clinical trials network’s PROMISE (The Promoting Maternal and Infant Survival Everywhere) study (NCT01061151) and the subset of mother-infant pairs who enrolled in the AP TDF exposure part of the nested P1084s substudy (NCT01066858). Both PROMISE and P1084s were registered on ClinicalTrials.gov on 10/02/2010, and the protocols are available online (https://www.impaactnetwork.org/). The full details of the PROMISE study design have also been described elsewhere [27]. Briefly, the AP Component was a randomized, open-label, strategy trial that compared the efficacy and safety of different ARV strategies to prevent HIV in utero and intrapartum vertical transmission in women living with HIV with CD4 cell count > 350 cells/mm^3^ in breastfeeding and formula feeding settings. Women were randomized at 14 weeks of pregnancy or later to one of three regimens: (1) zidovudine (ZDV) prophylaxis plus intrapartum single dose nevirapine (sdNVP)/TDF and emtricitabine (FTC) for 7 days (ZDV Alone); (2) ZDV, lamivudine, and lopinavir/ritonavir (ZDV-ART); (3) TDF, FTC, and lopinavir–ritonavir (TDF-ART). Under the first version of the trial protocol (Period 1), owing to limited safety data on TDF in pregnancy, only women positive for hepatitis B surface antigen (HBsAg) could be randomly assigned to TDF-ART; under the last version (Period 2), all women could be randomized 1:1:1 to the three regimens. Randomization was stratified by HBsAg status and country. All regimens were continued through 6 to 14 days postpartum. Infants received once-daily NVP prophylaxis in all trial groups, from birth through 6 weeks of age using birthweight-based dosing. Women remained in the AP Component through the Week 1 visit (6–14 days postpartum) and then, if eligible and willing, transitioned to a subsequent PROMISE study component, either the Postpartum (PP) Component or the Maternal Health (MH) Component, or continued follow-up in the AP Component observational follow-up (PROMISE study design is detailed in Additional file 1).

The P1084s substudy was a nested, comparative study of bone, renal, and growth outcomes. The maternal TDF versus no maternal TDF during pregnancy (or AP exposure) part of the P1084s substudy included women and their infants randomized in the AP Component of the PROMISE study; enrollment into the AP exposure part of the P1084s substudy occurred up to 21 days after AP study entry and before start of labor. The AP exposure part of the P1084s substudy started enrollment during PROMISE enrollment Period 1 that initially enrolled only HBsAg + women. During Period 2, all women enrolled in the AP Component of the PROMISE study from sites that could perform dual-energy X-ray absorptiometry (DXA) scans were eligible for the AP exposure part of the P1084s substudy.

### Trial sites and participants

The trial was conducted at 14 sites in seven countries (India, Malawi, South Africa, Tanzania, Uganda, Zambia, and Zimbabwe), and the AP Component enrolled between April 2011 and October 2014. When enrollment began, standard prevention of vertical transmission in this population was ZDV with intrapartum sdNVP and a 1-to-2-week “tail” of TDF/FTC to prevent maternal NVP resistance. Eligibility criteria included a CD4 count of at least 350 cells/mm^3^ (or a country-specific CD4 count threshold for initiating triple-drug ART, if that threshold was higher), gestation of at least 14 weeks and not in labor, no previous use of triple-drug ART, no clinical or immune-related indication for triple-drug ART, a hemoglobin level of at least 7.5 g/dL, an absolute neutrophil count of at least 750 cells/mm^3^, an alanine aminotransferase level of less than 2.5 times the upper limit of the normal range, an estimated creatinine clearance (CrCl) of over 60 mL/min, and no serious pregnancy complications. Key exclusion criteria were active tuberculosis (TB) or receipt of TB treatment within 30 days before trial entry, HBV infection requiring HBV treatment, a structural or conduction heart defect, or a fetus with a serious congenital malformation. All pregnant women provided written informed consent. The trial was approved by local and collaborating institutional review boards and reviewed every 6 months by an independent data and safety monitoring board.

### Trial procedures

Screening included confirmation of maternal HIV status, CD4 count measurement, and HBsAg status. Serum creatinine was measured on all women in the AP Component. For those women enrolled in the AP exposure part of the P1084s substudy, calcium and phosphate were measured at P1084s entry, delivery, 6, 26, and 74 weeks postpartum. For infants born to women enrolled in the AP exposure part of the P1084s substudy, serum creatinine, calcium, and phosphate were measured at birth, 10, 26, and 74 weeks of life. All women were to be followed until 96 weeks after the last woman in the AP Component of the PROMISE study delivered (estimated end of follow-up was 30 April 2017). However, on 7 July 2015 sites were instructed that all women in the PROMISE study should be informed of the Strategic Timing of Antiretroviral Therapy (START) study results [28] and that ART should be recommended for all women in the PROMISE study.

### Statistical analysis

Sample size calculations for the P1084s substudy were derived with a focus on DXA related outcome measures. Power calculations for CrCl outcome measures indicated more than adequate power for these measures, with sample sizes of < 145 per group for the scenarios considered in the protocol. Analyses were based on AP randomization and were carried out using an intent to treat approach (i.e., analyzed as randomized). The primary maternal outcome measure was calculated CrCl at Delivery, and the primary infant outcome measure was calculated CrCl at Birth. Secondary and additional maternal outcome measures of interest included change in calculated CrCl from Delivery to postpartum Week 6, Week 26, and Week 74 as well as calcium, phosphate, and hypophosphatemia at Delivery. Calcium and phosphate at Birth are included as additional infant outcome measures.

Maternal CrCl (mL/min) was calculated using the Cockroft-Gault equation, adjusted for female sex:

$$\left( {{14}0 - {\text{age }}\left[ {{\text{years}}} \right]} \right) \, \times \, ({\text{weight }}\left[ {{\text{kg}}} \right] \, \times \, 0.{85}/\left( {{72 } \times {\text{ Serum creatinine }}\left[ {{\text{mg}}/{\text{dL}}} \right]} \right),$$

with age at serum creatinine measurement and weight closest to day of serum creatinine measurement [29]. Infant CrCl was calculated using the revised Schwartz equation:

$$\left( {0.{413 } \times {\text{ height }}\left[ {{\text{cm}}} \right]} \right)/\left( {{\text{Serum creatinine }}\left[ {{\text{mg}}/{\text{dL}}} \right]} \right),$$

with age at serum creatinine measurement and height within one day of serum creatinine measurement [30]. Hypophosphatemia was defined as serum phosphate less than 2.5 mg/dL. Calculated CrCl grades were adapted from Version 2.0 (2.0 dated November 2014, 2.1 dated July 2017) of the Division of AIDS (DAIDS) Table for Grading the Severity of Adult and Pediatric Adverse Events [31]. Only the absolute value was used for grading purposes; percent change from baseline was not graded.

Maternal baseline values refer to the last determination before (but within 30 days) or on the AP randomization date. Gestational age at randomization was determined by the original obstetric clinical examination during pregnancy. All observations were censored on 6 July 2015, when the START study results were released [28].

The primary comparison was between the TDF-ART arm and the ZDV-ART arm, and the comparison between the TDF-ART arm and the ZDV Alone arm was considered secondary. Analyses that compared calculated CrCl between TDF-ART and ZDV-ART or ZDV Alone included HBsAg + women randomized during Period 1 and all women randomized during Period 2 (i.e., women eligible for TDF randomization), whereas analyses that compared calcium, phosphate, and hypophosphatemia pairwise between the three arms included women who were enrolled in the AP exposure part of the P1084s substudy. Additional analyses that compared calculated CrCl between women randomized to ZDV-ART and ZDV Alone included women randomized during both Period 1 and Period 2 and are presented in Additional file 2. Infant calculated CrCl, calcium, and phosphate were compared pairwise between the three arms using data from infants born to women enrolled in the AP exposure part of the P1084s study.

Two-sided Student’s t-tests assuming unequal variances compared arms at Delivery/Birth with respect to calculated CrCl. A two-sided P value of less than 0.05 indicated significance for the primary analyses, and there were no adjustments for multiple testing. Point estimates and two-sided 95% confidence intervals (CI) are presented for all comparisons. Comparisons of baseline data applied Wilcoxon/Kruskal–Wallis tests for continuous data and *X*^2^/exact tests for categorical data, as appropriate. All analyses were performed using SAS 9.4.

## Results

### Accrual and analysis exclusions

A total of 3543 women were randomized into the PROMISE AP Component between April 2011 and October 2014. Six women were excluded from analyses (two from the TDF-ART arm, two from the ZDV-ART arm, and two from the ZDV Alone arm). Three of these women were determined not to be pregnant at enrollment, two women had molar pregnancies, and one woman had an eligibility violation due to high blood pressure before enrollment and lost the pregnancy on the day of study entry, before taking the study drug. Overall, 1338 women eligible for TDF randomization were included in the primary analysis set.

**Fig. 1:**
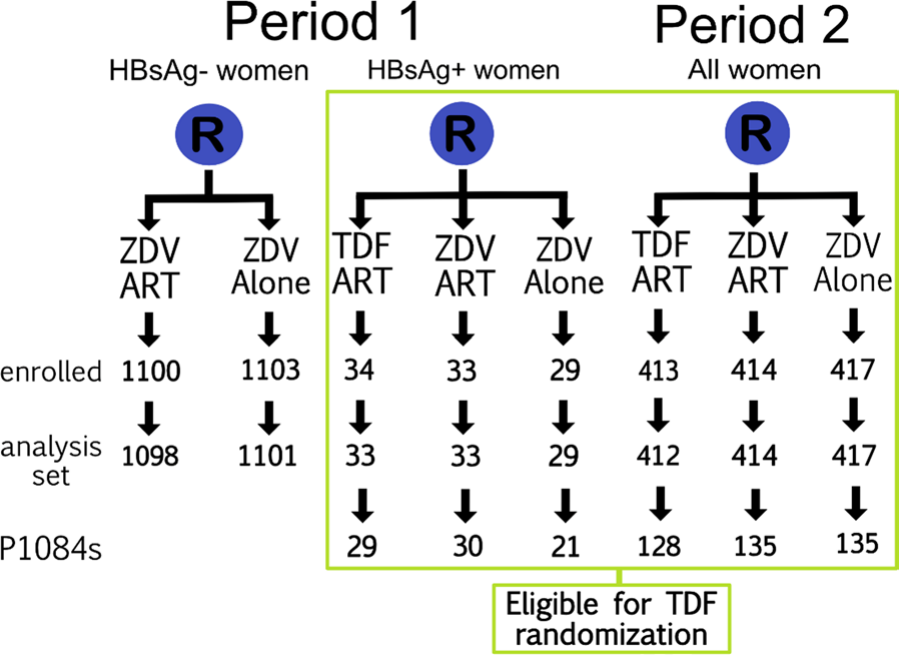
Enrollment and Analysis Inclusion by Antepartum Period, Hepatitis B Surface Antigen Status, and Randomization Arm. *HBsAg* Hepatitis B Surface Antigen, *ZDV* zidovudine, *TDF* tenofovir disoproxil fumarate, *ART* antiretroviral therapy

Of the women randomized in the PROMISE AP Component, 479 were co-enrolled in the AP exposure part of the P1084s substudy between July 2011 and December 2013, when the target sample size of 475 was reached. One woman was enrolled in the AP exposure part of the P1084s substudy in error and had no study visits. Per the pre-specified analysis plan, data from this woman were excluded from analysis on P1084s data. Enrollment and analysis inclusion of women by AP Period, HBsAg status, and AP randomization arm is shown in Fig. 1.

### Baseline characteristics

Maternal baseline characteristics at entry to the PROMISE AP Component for women eligible for TDF randomization are presented in Table 1. Among the 1338 women eligible for TDF randomization, less than 1% had a baseline calculated CrCl less than 80 mL/min, and one woman had a baseline value less than the entry criterion of 60 mL/min. This woman had a calculated CrCl greater than 60 mL/min at screening. For women enrolled in the P1084s substudy, no significant differences in baseline characteristic were detected across arms, except for age (P = 0.011) and weight (P = 0.046) (Additional file 3).

**Table 1:** Baseline Characteristics for Women Eligible for TDF Randomization

		TDF-ART (N = 445)	ZDV-ART (N = 447)	ZDV Alone (N = 446)	Total (N = 1338)
Age at randomization (years)	Median (Q1, Q3)	26.4 (22.7, 30.4)	26.6 (23.4, 30.3)	26.0 (22.3, 29.5)	26.4 (22.8, 30.1)
	18–< 30 years	326 (73)	324 (72)	344 (77)	994 (74)
	30–< 40 years	118 (27)	114 (26)	99 (22)	331 (25)
	40–< 50 years	1 (< 0.5)	9 (2)	3 (1)	13 (1)
Race	Black African	444 (≥ 99.5)	446 (≥ 99.5)	444 (≥ 99.5)	1334 (≥ 99.5)
	Indian	0 (0)	1 (< 0.5)	1 (< 0.5)	2 (< 0.5)
	Other	1 (< 0.5)	0 (0)	1 (< 0.5)	2 (< 0.5)
Country	South Africa	78 (18)	80 (18)	77 (17)	235 (18)
	Malawi	155 (35)	154 (34)	153 (34)	462 (35)
	Zambia	18 (4)	19 (4)	19 (4)	56 (4)
	Uganda	85 (19)	85 (19)	85 (19)	255 (19)
	Zimbabwe	99 (22)	96 (21)	99 (22)	294 (22)
	Tanzania	10 (2)	12 (3)	12 (3)	34 (3)
	India	0 (0)	1 (< 0.5)	1 (< 0.5)	2 (< 0.5)
AP Period	Period 1	33 (7)	33 (7)	29 (7)	95 (7)
	Period 2	412 (93)	414 (93)	417 (93)	1243 (93)
Weight (kg)	Median (Q1, Q3)	64.4 (58.0, 75.9)	64.0 (58.0, 73.0)	63.0 (57.0, 71.1)	63.8 (57.6, 73.3)
CD4 Cell Count (cells/mm^3^)	Median (Q1, Q3)	534.0 (432.0, 684.0)	540.0 (450.0, 660.0)	529.0 (431.0, 687.0)	536.0 (436.0, 680.0)
	< 350	2 (< 0.5)	2 (< 0.5)	0 (0)	4 (< 0.5)
	350–< 400	54 (12)	49 (11)	63 (14)	166 (12)
	400–< 450	82 (18)	59 (13)	81 (18)	222 (17)
	450–< 500	45 (10)	55 (12)	48 (11)	148 (11)
	500–< 750	182 (41)	208 (47)	182 (41)	572 (43)
	≥ 750	80 (18)	74 (17)	72 (16)	226 (17)
HIV RNA level (copies/mL)	N	445	447	444	1336
	Median (Q1, Q3)	8393.0 (1909.0, 28,454.0)	7003.0 (1510.0, 28,767.0)	6247.5 (1471.0, 24,133.0)	7339.5 (1604.0, 26,729.0)
	Below lower limit of quantification (LLQ) of the assay	19 (4)	21 (5)	10/444 (2)	50/1336 (4)
	< 400	26 (6)	33 (7)	45/444 (10)	104/1336 (8)
	400–1000	28 (6)	31 (7)	38/444 (9)	97/1336 (7)
	1000– < 10,000	165 (37)	170 (38)	165/444 (37)	500/1336 (37)
	10,000–< 100,000	170 (38)	163 (36)	153/444 (34)	486/1336 (36)
	100,000–< 200,000	24 (5)	14 (3)	17/444 (4)	55/1336 (4)
	≥ 200,000	13 (3)	15 (3)	16/444 (4)	44/1336 (3)
WHO Clinical Stage	Clinical stage I	434 (98)	436 (98)	431 (97)	1301 (97)
	Clinical stage II	11 (2)	11 (2)	15 (3)	37 (3)
HBsAg	Positive	48 (11)	48 (11)	42/445 (9)	138/1337 (10)
	Negative	397 (89)	399 (89)	403/445 (91)	1199/1337 (90)
Gestational age at Randomization (weeks)	N	445	446	446	1337
	Median (Q1, Q3)	26.0 (21.9, 31.1)	26.1 (21.3, 31.1)	26.1 (21.0, 31.1)	26.1 (21.3, 31.1)
	< 14	2 (< 0.5)	1/446 (< 0.5)	1 (< 0.5)	4/1337 (< 0.5)
	14–< 28	264 (59)	272/446 (61)	268 (60)	804/1337 (60)
	28–< 34	116 (26)	115/446 (26)	118 (26)	349/1337 (26)
	≥ 34	63 (14)	58/446 (13)	59 (13)	180/1337 (13)
Calculated CrCl (mL/min)	Median (Q1, Q3)	173.2 (141.7, 213.9)	168.0 (140.7, 201.7)	169.3 (141.6, 202.4)	170.1 (141.0, 206.1)
	> 50–60	1 (< 0.5)	0 (0)	0 (0)	1 (< 0.5)
	> 60–80	2 (< 0.5)	2 (< 0.5)	2 (< 0.5)	6 (< 0.5)
	> 80–100	5 (1)	14 (3)	10 (2)	29 (2)
	> 100–120	41 (9)	40 (9)	30 (7)	111 (8)
	> 120	396 (89)	391 (87)	404 (91)	1191 (89)

*TDF* tenofovir disoproxil fumarate, *ZDV* zidovudine, *Q1* 1st Quartile, *Q3* 3rd Quartile, *AP* antepartum, *HBsAg* hepatitis B surface antigen, *CrCl* creatinine clearance

For women eligible for TDF randomization, the overall median (25th, 75th percentile) follow-up time was 79.9 weeks (62.7, 100.4) (Additional file 4: Table S1). The median exposure time to TDF-based ART from randomization to Delivery was 11.7 weeks (5.9, 15.9) for the TDF-ART arm. For women in the ZDV-ART arm, the median exposure time to ZDV-based ART was 11.4 weeks (6.3, 16.6), and the median exposure time to ZDV was 12.1 weeks (7.1, 17.0) for the ZDV Alone arm. Among women eligible for TDF randomization, 92% of women in the TDF-ART arm were on a TDF-containing regimen at delivery, whereas only 2% and 6% in the other arms were on a TDF-containing regimen at delivery. By postpartum week 6, over 40% of women in each randomization arm were on a TDF-containing regimen. Approximately two thirds of the women in each arm were randomized with their infant in the PP Component to maternal TDF-ART or to infant NVP, and approximately 20% in the TDF-ART and ZDV-ART arms were randomized in the MH Component after delivery to continue or discontinue ART (Additional file 1: Fig. S1).

### Maternal outcome measures

#### Women eligible for TDF randomization

**Fig. 2:**
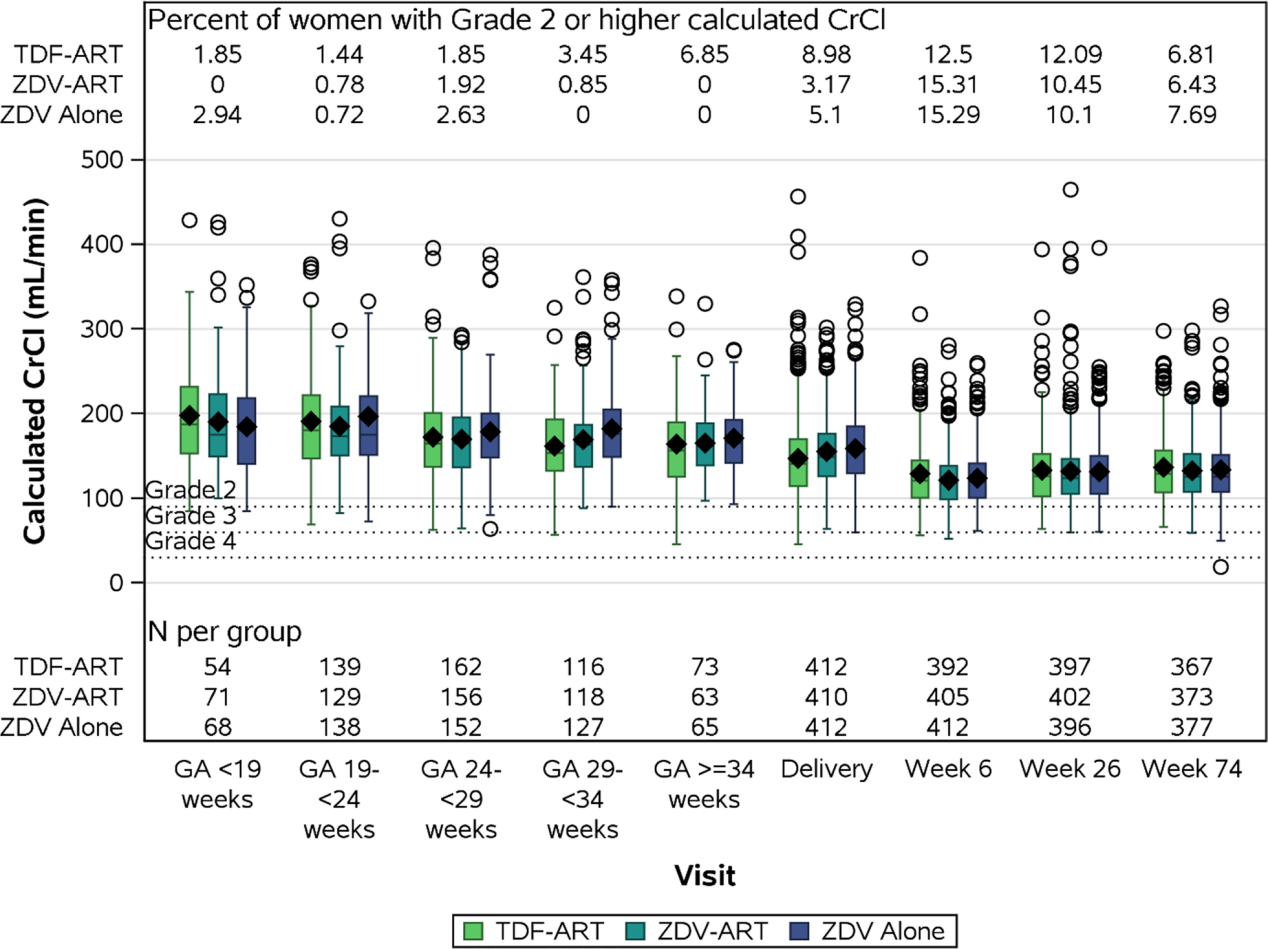
Distribution of Calculated CrCl across GA Categories, Delivery, and PP Study Visits*. *Among women eligible for TDF randomization; *CrCl* Creatinine Clearance, *ZDV* zidovudine, *TDF* tenofovir disoproxil fumarate, *GA* gestational age,.Diamonds represent mean calculated CrCl; CrCl (mL/min) was calculated using the Cockroft-Gault equation, adjusted for female sex [32]

Among women eligible for TDF randomization, the mean [standard deviation (sd)] calculated CrCl at Delivery was 147.0 mL/min (51.4) for the TDF-ART arm (n = 415), 155.0 mL/min (43.3) for the ZDV-ART arm (n = 417), and 158.5 mL/min (45.0) for the ZDV Alone arm (n = 446). Box plots of the distribution of calculated CrCl along with the percentage of women with a Grade 2 or higher calculated CrCl value are shown across gestational age categories and postpartum study visits in Fig. 2. As expected, calculated CrCl was elevated during pregnancy and delivery relative to postpartum visits.

In the primary comparison and outcome measure, the mean calculated CrCl at Delivery was lower in the TDF-ART arm than the ZDV-ART arm [mean difference (95% CI): − 8.0 mL/min (− 14.5, − 1.5), P = 0.015]. In secondary comparisons, the mean calculated CrCl at Delivery in the TDF-ART arm was also lower than the mean calculated CrCl at Delivery in the ZDV Alone arm [− 11.5 mL/min (− 18.0, − 4.9)]. The estimated mean differences between the TDF-ART arm and both the ZDV-ART arm and the ZDV Alone arm did not change substantially after adjusting for covariates and imputing missing values using a missing at random model (< 8% change from unadjusted treatment effect estimate; Additional file 5).

*Post-hoc* analyses that assessed change in calculated CrCl from Delivery by postpartum TDF exposure are summarized in Table 2. On average, the TDF-ART arm had smaller decreases in calculated CrCl from Delivery compared with both the ZDV-ART arm and ZDV Alone in women who had postpartum TDF exposure and in women who did not have postpartum TDF exposure across all study visits. However, women on a TDF-containing regimen at the postpartum visit had larger decreases in calculated CrCl from Delivery compared with women not on a TDF-containing regimen postpartum, on average. For example, at postpartum Week 6, the mean change in calculated CrCl for women in the TDF-ART arm who remained on a TDF-containing regimen was − 23.7 mL/min (− 29.6, − 17.8) compared with − 12.5 mL/min (-20.1, -5.0) for women who were no longer on a TDF-containing regimen. A similar pattern was observed within all three AP randomized arms across all time points.

**Table 2:** Change in Postpartum Calculated CrCl from Delivery by Postpartum TDF Exposure*

Postpartum TDF exposure	Change in Calculated CrCl (mL/min)	TDF-ART (N = 445)	ZDV-ART (N = 447)	ZDV alone (N = 446)
Week 6—delivery				
TDF	N	188	207	190
	Mean (95% CI)	− 23.7 (− 29.6, − 17.8)	− 38.6 (− 43.1, − 34.1)	− 38.6 (− 42.6, − 34.5)
	Mean difference (95% CI) from TDF-ART		14.9 (7.0, 22.8)	14.8 (6.7, 23.0)
No TDF	N	199	189	212
	Mean (95% CI)	− 12.5 (− 20.1, − 5.0)	− 27.0 (− 31.0, − 23.0)	− 31.7 (− 36.4, − 27.0)
	Mean difference (95% CI) from TDF-ART		14.5 (6.5, 22.5)	19.2 (11.3, 27.0)
Week 26—delivery				
TDF	N	192	206	195
	Mean (95% CI)	− 17.9 (− 24.8, − 11.1)	− 28.4 (− 34.5, − 22.4)	− 29.3 (− 33.3, − 25.3)
	Mean difference (95% CI) from TDF-ART		10.5 (2.1, 18.9)	11.4 (4.0, 18.7)
No TDF	N	193	184	188
	Mean (95% CI)	− 8.6 (− 12.8, − 4.3)	− 19.0 (− 25.7, − 12.3)	− 26.2 (− 31.6, − 20.9)
	Mean difference (95% CI) from TDF-ART		10.4 (1.8, 19.1)	17.7 (10.3, 25.1)
Week 24—delivery				
TDF	N	97	72	75
	Mean (95% CI)	− 8.4 (− 14.8, − 2.0)	− 30.7 (− 37.2, − 24.1)	− 32.2 (− 42.3, − 22.2)
	Mean difference (95% CI) from TDF-ART		22.3 (12.1, 32.5)	23.9 (12.0, 35.7)
No TDF	N	74	86	82
	Mean (95% CI)	− 5.2 (− 13.4, 3.0)	− 16.6 (− 24.6, − 8.6)	− 19.5 (− 29.6, − 9.5)
	Mean difference (95% CI) from TDF-ART		11.4 (1.0, 21.8)	14.3 (2.0, 26.6)
Week 6—delivery				
TDF	N	188	207	190
	Mean (95% CI)	− 23.7 (− 29.6, − 17.8)	− 38.6 (− 43.1, − 34.1)	− 38.6 (− 42.6, − 34.5)
	Mean difference (95% CI) from TDF-ART		14.9 (7.0, 22.8)	14.8 (6.7, 23.0)
No TDF	N	199	189	212
	Mean (95% CI)	− 12.5 (− 20.1, − 5.0)	− 27.0 (− 31.0, − 23.0)	− 31.7 (− 36.4, − 27.0)
	Mean difference (95% CI) from TDF-ART		14.5 (6.5, 22.5)	19.2 (11.3, 27.0)
Week 26—delivery				
TDF	N	192	206	195
	Mean (95% CI)	− 17.9 (− 24.8, − 11.1)	− 28.4 (− 34.5, − 22.4)	− 29.3 (− 33.3, − 25.3)
	Mean difference (95% CI) from TDF-ART		10.5 (2.1, 18.9)	11.4 (4.0, 18.7)
No TDF	N	193	184	188
	Mean (95% CI)	− 8.6 (− 12.8, − 4.3)	− 19.0 (− 25.7, − 12.3)	− 26.2 (− 31.6, − 20.9)
	Mean difference (95% CI) from TDF-ART		10.4 (1.8, 19.1)	17.7 (10.3, 25.1)
Week 74—delivery				
TDF	N	97	72	75
	Mean (95% CI)	− 8.4 (− 14.8, − 2.0)	− 30.7 (− 37.2, − 24.1)	− 32.2 (− 42.3, − 22.2)
	Mean difference (95% CI) from TDF-ART		22.3 (12.1, 32.5)	23.9 (12.0, 35.7)
No TDF	N	74	86	82
	Mean (95% CI)	− 5.2 (− 13.4, 3.0)	− 16.6 (− 24.6, − 8.6)	− 19.5 (− 29.6, − 9.5)
	Mean difference (95% CI) from TDF-ART		11.4 (1.0, 21.8)	14.3 (2.0, 26.6)

*Among women eligible for TDF randomization

*CrCl* creatinine clearance, *TDF* tenofovir disoproxil fumarate, *ZDV* zidovudine, *CI* confidence interval

#### Women randomized to either the ZDV-ART arm or the ZDV Alone arm during Periods 1 and 2

For women randomized to either the ZDV-ART arm or the ZDV Alone arm during Periods 1 and 2, the mean (sd) calculated CrCl at Delivery was 161.0 mL/min (50.5) for the ZDV-ART arm and 164.9 mL/min (48.7) for the ZDV Alone arm, and the mean calculated CrCl in the ZDV-ART arm was lower than the ZDV Alone arm [− 3.9 mL/min (− 7.4, − 0.3)] at Delivery (Additional file 2).

#### Women enrolled in the AP exposure part of the P1084s substudy

The summary statistics and pairwise mean differences of calcium, phosphate, and hypophosphatemia at Delivery are shown for women enrolled in the AP exposure part of the P1084s substudy in Table 3. Differences in mean calcium at delivery were close to 0 with narrow CIs that exclude a clinically relevant difference. Women in the TDF-ART arm had a lower mean phosphate at Delivery than both the ZDV-ART arm [mean difference (95% CI): − 0.14 mg/dL (− 0.28, − 0.01)] and the ZDV Alone arm [mean difference (95% CI): − 0.17 mg/dL (− 0.31, − 0.02)]. Although a greater percentage of women in the TDF-ART arm had hypophosphatemia at Delivery (4.23%) compared with both the ZDV-ART arm (1.38%) and the ZDV Alone arm (1.46%), the CIs for the mean differences included 0.

**Table 3:** Pairwise differences in calcium, phosphate, and hypophosphatemia at delivery for women in the P1084s substudy

		TDF-ART (N = 157)	ZDV-ART (N = 165)	ZDV Alone (N = 156)
Calcium (mg/dL)	N	141	143	137
	Mean (sd)	8.72 (0.60)	8.77 (0.66)	8.78 (0.59)
	Mean difference (95% CI) from TDF-ART		− 0.05 (− 0.20, 0.09)	− 0.06 (− 0.20, 0.08)
	Mean difference (95% CI) from ZDV-ART			− 0.01 (− 0.16, 0.14)
Phosphate (mg/dL)	N	142	145	137
	Mean (sd)	3.56 (0.60)	3.70 (0.58)	3.72 (0.61)
	Mean difference (95% CI) from TDF-ART		− 0.14 (− 0.28, − 0.01)	− 0.17 (− 0.31, − 0.02)
	Mean Difference (95% CI) from ZDV-ART			− 0.02 (− 0.16, 0.12)
Hypophosphatemia	No	136/142 (95.77)	143/145 (98.62)	135/137 (98.54)
	Yes	6/142 (4.23)	2/145 (1.38)	2/137 (1.46)
	Mean difference (95% CI) from TDF-ART		2.85 (− 0.97, 6.66)	2.77 (− 1.11, 6.64)
	Mean difference (95% CI) from ZDV-ART			− 0.08 (− 2.84, 2.68)

*CrCl* creatinine clearance, *TDF* tenofovir disoproxil fumarate, *ZDV* zidovudine, *sd* standard deviation, *CI*  confidence interval

### Infant outcome measures

Summary statistics and pairwise differences of calculated CrCl, calcium, and phosphate at Birth for infants born to women enrolled in the AP exposure part of the P1084s substudy are shown in Table 4. The mean (sd) calculated CrCl at Birth was 57.4 mL/min per 1.73 m^2^ (22.4) for the TDF-ART arm, 59.9 mL/min per 1.73 m^2^ (25.9) for the ZDV-ART arm, and 62.0 mL/min per 1.73 m^2^ (25.7) for the ZDV Alone arm. For the primary comparison, the mean calculated CrCl at Birth in the TDF-ART arm was not significantly different from the ZDV-ART arm [mean difference (95% CI): − 2.5 mL/min (− 8.4, 3.5), P = 0.42]. Differences in mean calcium and phosphate at Birth were also close to 0 with narrow CIs that exclude a clinically relevant differences.

**Table 4:** Pairwise differences in calculated creatinine clearance, calcium, and phosphate at birth for infants in P1084s

		TDF-ART (N = 157)	ZDV-ART (N = 165)	ZDV Alone (N = 156)
Calculated CrCl (mL/min per 1.73 m^2^)	N	126	128	115
	Mean (sd)	57.4 (22.4)	59.9 (25.9)	62.0 (25.7)
	Mean difference (95% CI) from TDF-ART		− 2.5 (− 8.4, 3.5)	− 4.6 (− 10.7, 1.6)
	P value*		0.42	
	Mean difference (95% CI) from ZDV-ART			− 2.1 (− 8.6, 4.4)
Calcium (mg/dL)	N	131	136	124
	Mean (sd)	10.30 (1.16)	10.26 (0.91)	10.30 (1.16)
	Mean difference (95% CI) from TDF-ART		0.04 (− 0.22, 0.29)	− 0.01 (− 0.29, 0.28)
	Mean difference (95% CI) from ZDV-ART			− 0.04 (− 0.30, 0.21)
Phosphate (mg/dL)	N	133	138	125
	Mean (sd)	6.28 (2.79)	6.12 (1.13)	7.32 (8.06)
	Mean difference (95% CI) from TDF-ART		0.16 (− 0.35, 0.68)	− 1.04 (− 2.54, 0.46)
	Mean difference (95% CI) from ZDV-ART			− 1.20 (− 2.64, 0.24)

*P value based on t-test assuming unequal variances

*CrCl* creatinine clearance, *TDF* tenofovir disoproxil fumarate, *ZDV* zidovudine, *sd* standard deviation, *CI*  confidence interval

## Discussion

The PROMISE trial assessed the efficacy and safety of different ARV strategies in prevention of perinatal HIV infection in a large number of women living with HIV. The three-arm design of the study encompassing TDF-ART, ZDV-ART, and ZDV Alone arms allowed for assessment of renal safety in pregnant women and their infants during the antenatal, delivery, and postpartum periods. In this primary analysis of the renal safety in the PROMISE study, we show that although maternal mean calculated CrCl at Delivery was lower in the TDF-ART than in the ZDV-ART and ZDV Alone arms, the difference between arms is unlikely to be clinically significant. In addition, *post-hoc* analyses showed that on average, the TDF-ART arm had smaller decreases in calculated CrCl from Delivery compared with both the ZDV-ART arm and ZDV Alone. Although the AP randomized treatment effect on maternal calculated CrCl change from Delivery did not differ by maternal postpartum TDF exposure, women with postpartum TDF exposure had a larger decrease in calculated CrCl from Delivery compared with women who did not. This pattern was consistent across all arms.

Although the definitions of renal toxicity vary, some studies report statistically significant renal function decline associated with TDF use. However, there is no consensus on the clinical significance of this effect [32–34]. Other studies have reported no statistical or clinical association of renal function decline in patients living with HIV receiving TDF [35–40].

Because limited studies have focused assessment of TDF use in pregnancy, there is little agreement on TDF effects in this population. Myer et al. [41] concluded that renal dysfunction in 238 pregnant women living with HIV was less common than in other adults living with HIV who were eligible for ART (1014 non-pregnant women and 609 men). The median serum creatinine in pregnant women (46 µmol/L) was lower and the median CrCl (163 ml/min/1.73 m^2^) was higher than other groups. Of note, compared with non-pregnant adults, eligible pregnant women were younger, in earlier stages of HIV disease, had higher CD4 cell counts, and had lower HIV viral loads [41].

For phosphate, women on the TDF-ART arm had lower mean phosphate at Delivery than both the ZDV-ART arm and the ZDV Alone arm. Although women in the TDF-ART arm were more likely to have hypophosphatemia at Delivery compared with both the ZDV-ART arm and the ZDV Alone arms, these differences were not statistically or clinically significant. In addition, differences in mean calcium at delivery were close to 0 with narrow CIs that exclude a clinically relevant difference. An observational study in 63 Vietnamese pregnant women with HIV also showed a tendency for higher levels of serum creatinine and lower concentrations of serum phosphorus in women on a TDF-based regimen compared with women on a ZDV-based regimen. However, these differences were also not significant [42].

For infants born to women enrolled in the AP exposure part of the P1084s substudy, calculated CrCl at Birth was not significantly different between arms, and differences in mean calcium and phosphate at Birth were also close to 0 with narrow CIs that exclude a clinically relevant differences. In the observational study mentioned above, maternal TDF use was also not associated with infant renal dysfunction [42].

Comparison of renal effects of TDF between studies is not straight forward because studies are heterogeneous in terms of the patient population, study type (i.e., cohort studies, retrospective studies, and randomized controlled studies), and outcomes measure of interest (for example, glycosuria, phosphaturia, CrCl, eGFR, serum creatinine, albumin creatinine ratio, protein creatinine ratio). Although TDF specifically affects proximal tubular dysfunction, some studies have not systematically used tests specific for proximal tubular dysfunction. This distinction may affect the significance of the reported renal effects. In clinical practice, some significant outcome measures may be surrogate outcomes that develop due to the disease process. However, clinical measurements outside of the normal range may not reflect significant clinical events, further complicating the interpretation of outcome measures often specified in studies. In our study, one woman randomized to the ZDV Alone arm had acute renal failure shortly before delivery. Another woman, also in the ZDV Alone arm, had acute renal insufficiency on the day of delivery. Both conditions were resolved about two weeks later. Similar cases were not reported in the TDF-ART arm. Of note, our study population was in good health at study enrollment. This may explain lack of clinical deterioration despite decrease in calculated CrCl in women who had antepartum and/or postpartum TDF exposure. We also did not assess urine protein-to-creatinine ratio, a more sensitive marker for proximal renal tubulopathy associated with TDF.

This study has several limitations. Approximately 25% of participants were not randomized as part of a subsequent PROMISE component following delivery. Although over half of the participants were on a postpartum TDF regimen and the distribution was similar across AP randomization arms, it is less clear how non-randomized TDF changes may influence changes in calculated CrCl after delivery. One infant in the TDF-ART arm had a serum creatinine measurement that was below the lower limit of quantification at birth. This value was set to missing in all analyses.

## Conclusions

Triple ART regimens that contain TDF form the mainstay of many national HIV treatment programs, and continued research to assess the safety of ART is recommended [27, 43]. During pregnancy, the TDF-ART regimen had no observed safety concerns for maternal renal function. Although limited in its scope, the analysis also demonstrated no renal dysfunction in infants born to mothers that used TDF-ART to prevent vertical HIV transmission.

## Supplementary Information

**Additional file 1:** PROMISE Study Design.

**Additional file 2: **Comparison of the ZDV ART and the ZDV Alone arms.

**Additional file 3: **Additional Baseline Characteristics.

**Additional file 4: Table S1. **PROMISE Study Follow-up Time and Tenofovir Disoproxil Fumarate (TDF) Exposure for Women Eligible for TDF Randomization.

**Additional file 5: **Supplementary and Sensitivity Analyses.

## Data Availability

The data cannot be made publicly available due to the ethical restrictions in the study’s informed consent documents and in the International Maternal Pediatric Adolescent AIDS Clinical Trials (IMPAACT) Network’s approved human subjects protection plan; public availability may compromise participant confidentiality. However, data are available to all interested researchers upon request to the IMPAACT Statistical and Data Management Center’s data access committee by email to gro.frtsf@atad.cads or ude.dravrah@atad.cads. This committee reviews and responds to requests for data, obtains necessary approvals from IMPAACT leadership and the National Institutes of Health (NIH), arranges for signature of a Data Use Agreement, and releases the requested data.

## Abbreviations

AP: Antepartum
ART: Antiretroviral therapy
ARV: Antiretroviral
CI: Confidence intervals
DXA: Dual-energy X-ray absorptiometry
eGFR: Estimated glomerular filtration rate
FDA: Food and Drug Administration
FTC: Emtricitabine
HBV: Hepatitis B virus
MH: Maternal health
PP: Postpartum
PrEP: Prevention as pre-exposure prophylaxis
PROMISE: Promoting Maternal and Infant Survival Everywhere
sd: Standard deviation
sdNVP: Single dose nevirapine
START: Strategic Timing of Antiretroviral Therapy
TB: Tuberculosis
TDF: Tenofovir disoproxil fumarate
UK: United Kingdom
US: United States
WHO: World Health Organization
ZDV: Zidovudine
