# Supplementary material for: Maternal and infant renal safety following tenofovir disoproxil fumarate exposure during pregnancy in a randomized control trial
Source: BMC Infect Dis. 2022 Jul 20;22:634. doi: 10.1186/s12879-022-07608-8 (PMC9297643; doi:10.1186/s12879-022-07608-8)
Supplement: Supplementary file 1 — Additional file 1: PROMISE Study Design. [file 12879_2022_7608_MOESM1_ESM.docx]

The antepartum (AP) Component of PROMISE was a randomized, open-label, strategy trial that compared the efficacy and safety of different antiretroviral (ARV) strategies to prevent HIV *in utero* and intrapartum vertical transmission in women living with HIV with CD4 cell count > 350 cells/mm^3^ in breastfeeding and formula feeding settings. Women were randomized at 14 weeks of pregnancy or later to one of three regimens: 1) zidovudine (ZDV) prophylaxis plus intrapartum single dose nevirapine (sdNVP)/tenofovir disoproxil fumarate (TDF) and emtricitabine (FTC) for 7 days (ZDV Alone); 2) ZDV, lamivudine, and lopinavir/ritonavir (ZDV-ART); 3) TDF, FTC, and lopinavir–ritonavir (TDF-ART). Under the first version of the trial protocol (Period 1), owing to limited safety data on TDF in pregnancy, only women positive for hepatitis B surface antigen (HBsAg) could be randomly assigned to TDF-ART; under the last version (Period 2), all women could be randomized 1:1:1 to the three regimens. Randomization was stratified by HBsAg status and country. All regimens were continued through 6 to 14 days postpartum. Infants received once-daily NVP prophylaxis in all trial groups, from birth through 6 weeks of age using birthweight-based dosing.

Women remained in the AP Component through the Week 1 visit (6-14 days postpartum) and then, if eligible and willing, transitioned to a subsequent PROMISE study component, either the Postpartum (PP) Component or the Maternal Health (MH) Component, or continued follow-up in the AP Component observational follow-up. The PP Component randomized eligible mother-infant pairs to initiate maternal TDF-ART or to continue infant NVP prophylaxis beyond 6 weeks. The MH Component enrolled women randomized to receive either ZDV-ART or TDF-ART in the AP Component and chose not to breastfeed. Following enrollment in the MH Component, women were randomized to either continue or discontinue the study triple ARV regimen (ZDV-ART or TDF-ART). Women who were ineligible or declined participation in the PP and the MH Components were followed as part of the AP Component observational follow-up.


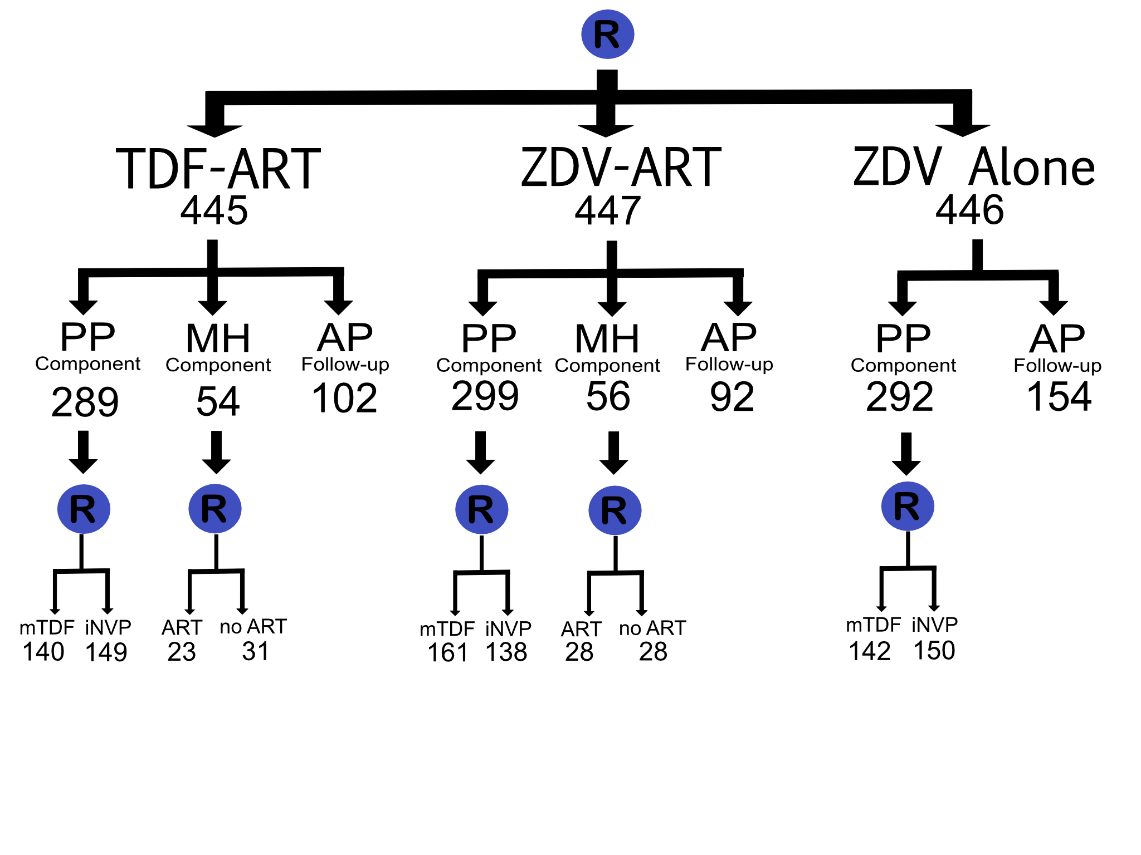


**Additional File 1 Figure 1**. Enrollment of Women into Subsequent PROMISE Study Components by Antepartum Randomization Arm for Women Eligible for Tenofovir Disoproxil Fumarate Randomization. AP = Antepartum; PP = Postpartum; MH = Maternal Health ZDV = zidovudine; TDF = tenofovir disoproxil fumarate; ART = antiretroviral therapy; iNVP = infant nevirapine
