# Supplementary material for: Maternal and infant renal safety following tenofovir disoproxil fumarate exposure during pregnancy in a randomized control trial
Source: BMC Infect Dis. 2022 Jul 20;22:634. doi: 10.1186/s12879-022-07608-8 (PMC9297643; doi:10.1186/s12879-022-07608-8)
Supplement: Supplementary file 2 — Additional file 2: Comparison of the ZDV ART and the ZDV Alone arms. [file 12879_2022_7608_MOESM2_ESM.docx]

Analyses that compared calculated CrCl between women randomized to ZDV-ART and ZDV Alone included women randomized during both Period 1 and Period 2. Overall, 3092 women were randomized to either the ZDV-ART arm or the ZDV Alone arm during Periods 1 and 2. Baseline characteristics were similar to women eligible for TDF randomization, except for the smaller percentage (3% compared to 10%) of HBsAg+ women as a result of the study design where initially (Period 1) only HBsAg+ women were eligible for randomization to the TDF-ART arm (Additional File 2 Table 1). Additional File 2 Table 2 summarizes the PROMISE study follow-up and study treatment exposure, and Additional File 2 Table 3 provides the pairwise differences in calculated CrCl at Delivery and change in calculated CrCl from Delivery by TDF exposure for women randomized during Periods 1 and 2 to ZDV-ART or ZDV alone. The distribution of calculated CrCl across GA categories, Delivery, and PP study visits for women randomized during Periods 1 and 2 to ZDV-ART or ZDV Alone is shown in Additional File 2 Figure 1. For women randomized to either the ZDV-ART arm or the ZDV Alone arm during Periods 1 and 2, the mean (sd) calculated CrCl at Delivery was 161.0 mL/min (50.5) for the ZDV-ART arm and 164.9 mL/min (48.7) for the ZDV Alone arm, and the mean calculated CrCl in the ZDV-ART arm was lower than the ZDV Alone arm [-3.9 mL/min (-7.4, -0.3)] at Delivery (Additional File 2).

**Additional File 2 Table 1.** Baseline Characteristics for Women Randomized during Periods 1 and 2 to Zidovudine (ZDV) ART or ZDV Alone.

|  | | **ZDV-ART (N=1545)** | **ZDV Alone (N=1547)** | **Total (N=3092)** |
| --- | --- | --- | --- | --- |
| Age at randomization (years) | N | 1545 | 1547 | 3092 |
|  | Median (Q1, Q3) | 26.6 (23.3, 30.3) | 26.5 (22.8, 30.1) | 26.5 (23.0, 30.2) |
|  |  |  |  |  |
|  | 18 - <30 years | 1130 (73) | 1148 (74) | 2278 (74) |
|  | 30 - <40 years | 391 (25) | 384 (25) | 775 (25) |
|  | 40 - <50 years | 24 (2) | 15 (1) | 39 (1) |
|  |  |  |  |  |
| Race | Black African | 1498 (97) | 1499 (97) | 2997 (97) |
|  | Indian | 46 (3) | 46 (3) | 92 (3) |
|  | Other | 1 (<0.5) | 2 (<0.5) | 3 (<0.5) |
|  |  |  |  |  |
| Country | South Africa | 510 (33) | 513 (33) | 1023 (33) |
|  | Malawi | 493 (32) | 489 (32) | 982 (32) |
|  | Zambia | 31 (2) | 32 (2) | 63 (2) |
|  | Uganda | 205 (13) | 207 (13) | 412 (13) |
|  | Zimbabwe | 236 (15) | 237 (15) | 473 (15) |
|  | Tanzania | 24 (2) | 23 (1) | 47 (2) |
|  | India | 46 (3) | 46 (3) | 92 (3) |
|  |  |  |  |  |
| AP Period | Period 1 | 1131 (73) | 1130 (73) | 2261 (73) |
|  | Period 2 | 414 (27) | 417 (27) | 831 (27) |
|  |  |  |  |  |
| Weight (kg) | N | 1545 | 1547 | 3092 |
|  | Median (Q1, Q3) | 64.6 (58.0, 74.1) | 64.0 (57.5, 73.5) | 64.2 (57.8, 74.0) |
|  |  |  |  |  |
| CD4 Cell Count (cells/mm^3^) | N | 1544 | 1547 | 3091 |
|  | Median (Q1, Q3) | 526.0 (440.0, 650.5) | 537.0 (435.0, 672.0) | 531.0 (437.0, 661.0) |
|  |  |  |  |  |
|  | < 350 | 31/1544 (2) | 27 (2) | 58/3091 (2) |
|  | 350 - < 400 | 188/1544 (12) | 204 (13) | 392/3091 (13) |
|  | 400 - < 450 | 218/1544 (14) | 231 (15) | 449/3091 (15) |
|  | 450 - < 500 | 224/1544 (15) | 182 (12) | 406/3091 (13) |
|  | 500 - < 750 | 650/1544 (42) | 659 (43) | 1309/3091 (42) |
|  | ≥ 750 | 233/1544 (15) | 244 (16) | 477/3091 (15) |
|  |  |  |  |  |
| HIV RNA level (copies/mL) | N | 1542 | 1540 | 3082 |
|  | Median (Q1, Q3) | 7339.5 (1604.0, 26729.0) | 8002.0 (1854.0, 28767.0) | 6409.0 (1450.5, 22720.0) |
|  |  |  |  |  |
|  | Below lower limit of quantification (LLQ) of the assay | 81/1542 (5) | 67/1540 (4) | 148/3082 (5) |
|  | <400 | 92/1542 (6) | 148/1540 (10) | 240/3082 (8) |
|  | 400 - 1000 | 105/1542 (7) | 115/1540 (7) | 220/3082 (7) |
|  | 1000- <10000 | 574/1542 (37) | 579/1540 (38) | 1153/3082 (37) |
|  | 10000 - <100000 | 570/1542 (37) | 532/1540 (35) | 1102/3082 (36) |
|  | 100000 - <200000 | 71/1542 (5) | 60/1540 (4) | 131/3082 (4) |
|  | ≥200000 | 49/1542 (3) | 39/1540 (3) | 88/3082 (3) |
|  |  |  |  |  |
| WHO Clinical Stage | Clinical stage I | 1506/1542 (98) | 1493/1544 (97) | 2999/3086 (97) |
|  | Clinical stage II | 34/1542 (2) | 50/1544 (3) | 84/3086 (3) |
|  | Clinical stage III | 2/1542 (<0.5) | 1/1544 (<0.5) | 3/3086 (<0.5) |
|  |  |  |  |  |
| HBsAg | Positive | 48 (3) | 42 (3) | 90/3090 (3) |
|  | Negative | 1497 (97) | 1503 (97) | 3000/3090 (97) |
|  |  |  |  |  |
| Gestational age at Randomization (weeks) | N | 1544 | 1547 | 3091 |
|  | Median (Q1, Q3) | 25.2 (20.6, 30.1) | 25.7 (20.9, 30.3) | 25.4 (20.7, 30.1) |
|  |  |  |  |  |
|  | < 14 | 8/1544 (1) | 10 (1) | 18/3091 (1) |
|  | 14 - < 28 | 1008/1544 (65) | 956 (62) | 1964/3091 (64) |
|  | 28 - < 34 | 368/1544 (24) | 418 (27) | 786/3091 (25) |
|  | ≥ 34 | 160/1544 (10) | 163 (11) | 323/3091 (10) |
|  |  |  |  |  |
| Calculated CrCl (mL/min) | N | 1545 | 1547 | 3092 |
|  | Median (Q1, Q3) | 179.4 (145.8, 219.5) | 178.2 (148.6, 218.3) | 179.0 (147.8, 218.8) |
|  |  |  |  |  |
|  | >50 - 60 | 0 (0) | 1 (<0.5) | 1 (<0.5) |
|  | >60 - 80 | 7 (<0.5) | 6 (<0.5) | 13 (<0.5) |
|  | >80 - 100 | 30 (2) | 27 (2) | 57 (2) |
|  | >100 - 120 | 107 (7) | 88 (6) | 195 (6) |
|  | > 120 | 1401 (91) | 1425 (92) | 2826 (91) |

ZDV = zidovudine; Q1 = 1^st^ Quartile; Q3 = 3^rd^ Quartile; AP = Antepartum; HBsAg = Hepatitis B Surface Antigen; CrCl = creatinine clearance.

**Additional File 2 Table 2.** PROMISE Study Follow-up Time and Tenofovir Disoproxil Fumarate Exposure for Women Randomized during Periods 1 and 2 to Zidovudine (ZDV) ART or ZDV Alone.

|  |  | **ZDV-ART (N=1545)** | **ZDV Alone (N=1547)** | **Total (N=3092)** |
| --- | --- | --- | --- | --- |
| Follow-up at START Study Censor Date (Weeks) | N | 1545 | 1547 | 3092 |
|  | Median (Q1, Q3) | 137.9 (86.7, 171.6) | 139.9 (88.7, 171.9) | 138.7 (87.9, 171.7) |
|  |  |  |  |  |
| On Study at Start of Week 74 Window | Yes | 1223 (79) | 1234 (80) | 2457 (79) |
|  | No | 322 (21) | 313 (20) | 635 (21) |
|  |  |  |  |  |
| Time on Regimen from Randomization to Delivery (Weeks) | N | 1511 | 1506 | 3017 |
|  | Median (Q1, Q3) | 12.4 (7.1, 17.1) | 12.6 (8.0, 17.6) | 12.5 (7.6, 17.4) |
|  |  |  |  |  |
| **Study Visit** | **On TDF-Containing Regimen*** |  |  |  |
| Delivery | No | 1486/1504 (99) | 1465/1516 (97) | 2951/3020 (98) |
|  | Yes | 18/1504 (1) | 51/1516 (3) | 69/3020 (2) |
|  |  |  |  |  |
| Week 6 | No | 849/1494 (57) | 929/1511 (61) | 1778/3005 (59) |
|  | Yes | 645/1494 (43) | 582/1511 (39) | 1227/3005 (41) |
|  |  |  |  |  |
| Week 26 | No | 831/1452 (57) | 894/1463 (61) | 1725/2915 (59) |
|  | Yes | 621/1452 (43) | 569/1463 (39) | 1190/2915 (41) |
|  |  |  |  |  |
| Week 74 | No | 753/1152 (65) | 818/1162 (70) | 1571/2314 (68) |
|  | Yes | 399/1152 (35) | 344/1162 (30) | 743/2314 (32) |
| *Excluding TDF tail  TDF = tenofovir disoproxil fumarate; ZDV = zidovudine; Q1 = 1^st^ Quartile; Q3 = 3^rd^ Quartile | | | | |

**Additional File 2 Table 3.** Pairwise Difference in Calculated Creatinine Clearance (CrCl) at Delivery and Change in Calculated CrCl from Delivery by Tenofovir Disoproxil Fumarate Exposure for Women Randomized during Periods 1 and 2 to Zidovudine (ZDV) ART or ZDV Alone.

|  | **ZDV-ART (N=1545)** | | **ZDV Alone (N=1547)** | | **ZDV-ART - ZDV Alone** |
| --- | --- | --- | --- | --- | --- |
| **Postpartum TDF Exposure** | **Mean (95% CI)** | **N** | **Mean (95% CI)** | **N** | **Mean Difference (95% CI)** |
|  |  |  |  |  |  |
| **Delivery** | 161.0 (158.4, 163.6) | 1466 | 164.9 (162.4, 167.4) | 1476 | -3.9 (-7.4, -0.3) |
|  |  |  |  |  |  |
| **Week 6 - Delivery** |  |  |  |  |  |
| TDF | -38.0 (-41.5, -34.5) | 639 | -45.2 (-48.0, -42.4) | 579 | 7.2 (2.7, 11.7) |
| no TDF | -28.1 (-30.5, -25.7) | 770 | -31.9 (-34.9, -28.9) | 854 | 3.8 (-0.1, 7.7) |
|  |  |  |  |  |  |
| **Week 26 - Delivery** |  |  |  |  |  |
| TDF | -30.8 (-34.4, -27.2) | 614 | -32.1 (-35.1, -29.2) | 564 | 1.3 (-3.4, 6.1) |
| no TDF | -22.9 (-25.8, -20.0) | 744 | -26.0 (-29.0, -22.9) | 805 | 3.1 (-1.1, 7.2) |
|  |  |  |  |  |  |
| **Week 74 - Delivery** |  |  |  |  |  |
| TDF | -27.7 (-32.3, -23.1) | 392 | -31.3 (-35.8, -26.9) | 338 | 3.7 (-3.5, 10.9) |
| no TDF | -20.2 (-23.8, -16.6) | 640 | -23.9 (-28.2, -19.7) | 690 | 3.7 (-1.6, 9.0) |
| CrCl = creatinine clearance; ZDV = zidovudine; CI = confidence interval | | | | | |


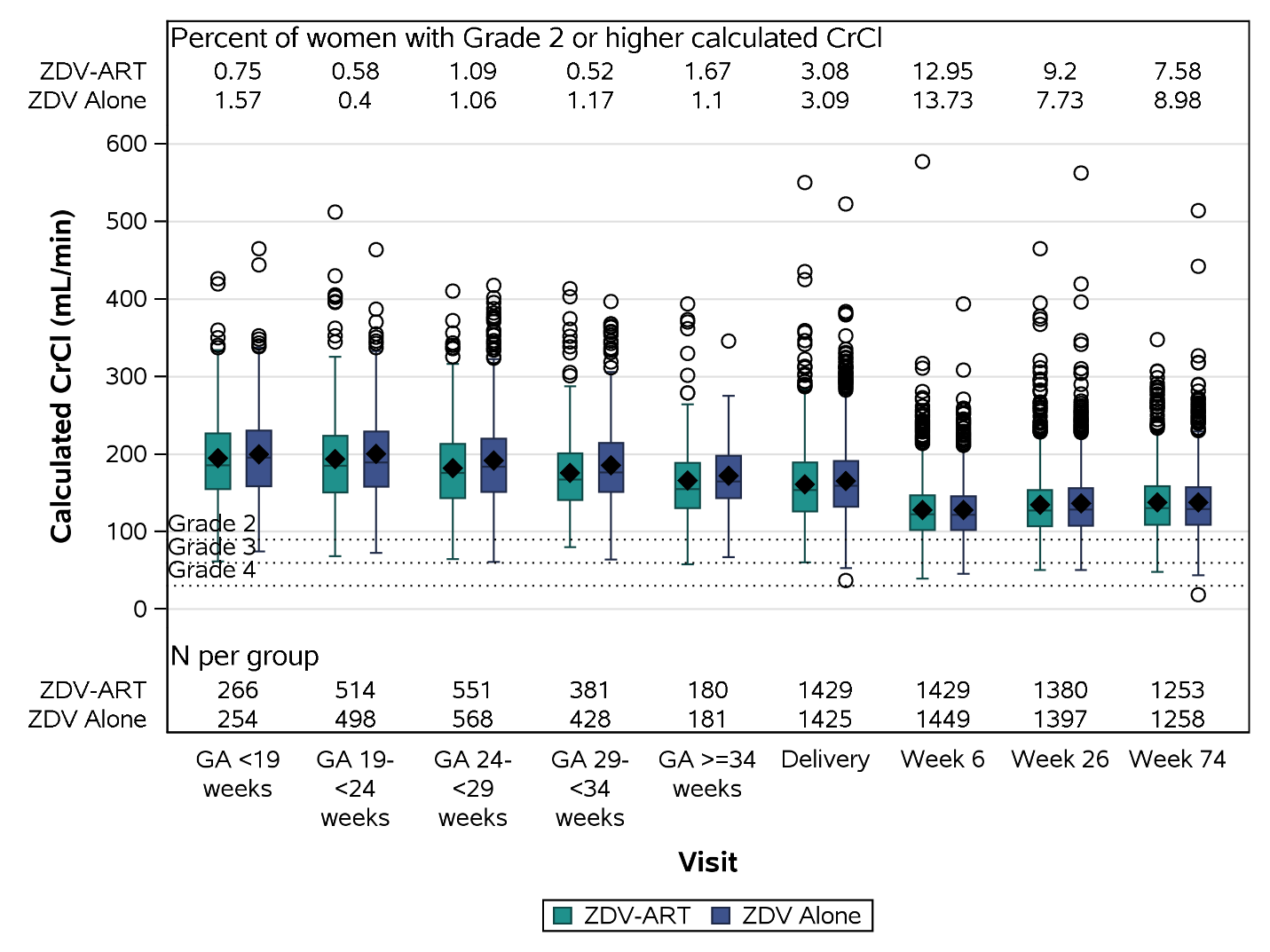


**Additional File 2 Figure 1.** Distribution of Calculated Creatinine Clearance (CrCl) across Gestational Age (GA) Categories, Delivery, and Postpartum (PP) Study Visits for Women Randomized during Periods 1 and 2 to Zidovudine (ZDV) ART or ZDV Alone.

CrCl = Creatinine Clearance; ZDV = zidovudine; GA = gestational age; Diamonds represent mean calculated CrCl; CrCl (mL/min) was calculated using the Cockroft-Gault equation, adjusted for female sex [32].
