# Supplementary material for: Maternal and infant renal safety following tenofovir disoproxil fumarate exposure during pregnancy in a randomized control trial
Source: BMC Infect Dis. 2022 Jul 20;22:634. doi: 10.1186/s12879-022-07608-8 (PMC9297643; doi:10.1186/s12879-022-07608-8)
Supplement: Supplementary file 3 — Additional file 3: Additional Baseline Characteristics. [file 12879_2022_7608_MOESM3_ESM.docx]

Comparisons of baseline data applied Wilcoxon/Kruskal-Wallis tests for continuous data and *X*^2^/exact tests for categorical data, as appropriate. As shown in Additional File 3 Table 1, except for age (P = 0.011) and weight (P = 0.046), no significant differences in baseline characteristic were detected across arms for the P1084s substudy. Only country at enrollment was significantly different between the 478 women who enrolled and analyzed in the substudy and the 97 women who did not enroll (Additional File 3 Table 2).

**Additional File Table 1.** Baseline Characteristics of Women Enrolled in the Antepartum Exposure Part of the P1084s Substudy.

|  | | **TDF-ART (N=157)** | **ZDV-ART (N=165)** | **ZDV Alone (N=156)** | **Total (N=478)** | **P Value** |
| --- | --- | --- | --- | --- | --- | --- |
|  | | | | | | |
| Age at randomization (years) | N | 157 | 165 | 156 | 478 | 0.011* |
|  | Median (Q1, Q3) | 27.1 (23.9, 30.9) | 27.4 (24.0, 30.8) | 25.8 (22.8, 29.2) | 26.7 (23.3, 30.2) |  |
|  | | | | | | |
|  | 18 - <30 years | 109 (69) | 118 (72) | 124 (79) | 351 (73) | 0.07** |
|  | 30 - <40 years | 47 (30) | 43 (26) | 32 (21) | 122 (26) |  |
|  | 40 - <50 years | 1 (1) | 4 (2) | 0 (0) | 5 (1) |  |
|  | | | | | | |
| Race | Black African | 156 (99) | 165 (100) | 156 (100) | 477 (≥99.5) |  |
|  | Other | 1 (1) | 0 (0) | 0 (0) | 1 (<0.5) |  |
|  | | | | | | |
| Country | South Africa | 26 (17) | 33 (20) | 24 (15) | 83 (17) | 0.95** |
|  | Malawi | 26 (17) | 28 (17) | 29 (19) | 83 (17) |  |
|  | Uganda | 47 (30) | 44 (27) | 45 (29) | 136 (28) |  |
|  | Zimbabwe | 58 (37) | 60 (36) | 58 (37) | 176 (37) |  |
|  | | | | | | |
| AP Period | Period 1 | 29 (18) | 30 (18) | 21 (13) | 80 (17) | 0.41** |
|  | Period 2 | 128 (82) | 135 (82) | 135 (87) | 398 (83) |  |
|  | | | | | | |
| Weight (kg) | N | 157 | 165 | 156 | 478 | 0.046* |
|  | Median (Q1, Q3) | 65.0 (59.0, 77.1) | 66.5 (60.4, 75.0) | 63.8 (57.2, 71.6) | 65.0 (58.7, 75.0) |  |
|  | | | | | | |
| CD4 Cell Count (cells/mm^3^) | N | 157 | 165 | 156 | 478 | 0.65* |
|  | Median (Q1, Q3) | 553.0 (439.0, 684.0) | 549.0 (441.0, 674.0) | 520.5 (422.5, 689.5) | 544.0 (434.0, 684.0) |  |
|  | | | | | | |
|  | < 350 | 2 (1) | 2 (1) | 0 (0) | 4 (1) | 0.75** |
|  | 350 - < 400 | 18 (11) | 19 (12) | 26 (17) | 63 (13) |  |
|  | 400 - < 450 | 23 (15) | 24 (15) | 28 (18) | 75 (16) |  |
|  | 450 - < 500 | 19 (12) | 15 (9) | 16 (10) | 50 (10) |  |
|  | 500 - < 750 | 69 (44) | 79 (48) | 63 (40) | 211 (44) |  |
|  | ≥ 750 | 26 (17) | 26 (16) | 23 (15) | 75 (16) |  |
|  | | | | | | |
| HIV RNA level (copies/mL) | N | 157 | 165 | 156 | 478 | 0.35* |
|  | Median (Q1, Q3) | 9736.0 (2036.0, 39171.0) | 7328.0 (1507.0, 31746.0) | 6866.5 (1471.0, 25266.0) | 8142.0 (1569.0, 31663.0) |  |
|  | | | | | | |
|  | Below lower limit of quantification (LLQ) of the assay | 9 (6) | 13 (8) | 2 (1) | 24 (5) | 0.25** |
|  | <400 | 6 (4) | 9 (5) | 11 (7) | 26 (5) |  |
|  | 400 - 1000 | 10 (6) | 11 (7) | 14 (9) | 35 (7) |  |
|  | 1000- <10000 | 54 (34) | 55 (33) | 62 (40) | 171 (36) |  |
|  | 10000 - <100000 | 61 (39) | 64 (39) | 50 (32) | 175 (37) |  |
|  | 100000 - <200000 | 10 (6) | 5 (3) | 11 (7) | 26 (5) |  |
|  | ≥200000 | 7 (4) | 8 (5) | 6 (4) | 21 (4) |  |
|  | | | | | | |
| WHO Clinical Stage | Clinical stage I | 153 (97) | 159 (96) | 149 (96) | 461 (96) | 0.64** |
|  | Clinical stage II | 4 (3) | 6 (4) | 7 (4) | 17 (4) |  |
|  | | | | | | |
| HBsAg | Positive | 32 (20) | 35 (21) | 24 (15) | 91 (19) | 0.36** |
|  | Negative | 125 (80) | 130 (79) | 132 (85) | 387 (81) |  |
|  | | | | | | |
| Gestational age at Randomization (weeks) | N | 157 | 165 | 156 | 478 | 0.29* |
|  | Median (Q1, Q3) | 27.3 (22.9, 33.3) | 27.0 (22.0, 32.0) | 26.4 (21.1, 31.4) | 27.0 (21.9, 32.0) |  |
|  | | | | | | |
|  | < 14 | 1 (1) | 1 (1) | 1 (1) | 3 (1) | 0.90** |
|  | 14 - < 28 | 82 (52) | 88 (53) | 89 (57) | 259 (54) |  |
|  | 28 - < 34 | 43 (27) | 50 (30) | 44 (28) | 137 (29) |  |
|  | ≥ 34 | 31 (20) | 26 (16) | 22 (14) | 79 (17) |  |
|  |  |  |  |  |  |  |
| Calculated CrCl (mL/min) | N | 157 | 165 | 156 | 478 | 0.44* |
|  | Median (Q1, Q3) | 176.4 (147.6, 214.6) | 175.0 (147.1, 213.3) | 169.6 (146.1, 200.4) | 172.6 (146.3, 211.0) |  |
|  | | | | | | |
|  | >60 - 80 | 0 (0) | 1 (1) | 1 (1) | 2 (<0.5) | 0.23** |
|  | >80 - 100 | 0 (0) | 3 (2) | 1 (1) | 4 (1) |  |
|  | >100 - 120 | 14 (9) | 14 (8) | 6 (4) | 34 (7) |  |
|  | > 120 | 143 (91) | 147 (89) | 148 (95) | 438 (92) |  |
|  | | | | | | |
| Calcium (mg/dL) | N | 105 | 112 | 101 | 318 | 0.92* |
|  | Median (Q1, Q3) | 8.8 (8.4, 9.2) | 8.8 (8.4, 9.1) | 8.8 (8.4, 9.1) | 8.8 (8.4, 9.1) |  |
|  | | | | | | |
| Phosphate (mg/dL) | N | 104 | 110 | 102 | 316 | 0.14* |
|  | Median (Q1, Q3) | 3.7 (3.2, 4.1) | 3.6 (3.1, 3.8) | 3.5 (3.2, 3.8) | 3.6 (3.1, 3.9) |  |
|  | | | | | | |
|  | 1.0-<1.4 | 0/104 (0) | 0/110 (0) | 1/102 (1) | 1/316 (<0.5) | 0.35** |
|  | ≥2 | 104/104 (100) | 110/110 (100) | 101/102 (99) | 315/316 (≥99.5) |  |
| *Kruskal-Wallis Test **Chi-Square/ Fishers Exact Test  TDF = tenofovir disoproxil fumarate; ZDV = zidovudine; Q1 = 1^st^ Quartile; Q3 = 3^rd^ Quartile; AP = Antepartum; HBsAg = Hepatitis B Surface Antigen; CrCl = creatinine clearance; | | | | | | |

**Additional File 3 Table 2.** Baseline Characteristics of Women Eligible for the Antepartum Exposure Part of the P1084s Substudy.

|  | | **Enrolled (N=478)** | **Not Enrolled (N=97)** | **Total (N=575)** | **P Value** |
| --- | --- | --- | --- | --- | --- |
| Age at randomization (years) | N | 478 | 97 | 575 | 0.97* |
|  | Median (Q1, Q3) | 26.7 (23.3, 30.2) | 26.9 (22.8, 30.8) | 26.7 (23.2, 30.2) |  |
|  |  |  |  |  |  |
|  | 18 - <30 years | 351 (73) | 69 (71) | 420 (73) | 0.29** |
|  | 30 - <40 years | 122 (26) | 25 (26) | 147 (26) |  |
|  | 40 - <50 years | 5 (1) | 3 (3) | 8 (1) |  |
|  |  |  |  |  |  |
| Race | Black African | 477 (≥99.5) | 96 (99) | 573 (≥99.5) |  |
|  | Other | 1 (<0.5) | 1 (1) | 2 (<0.5) |  |
|  |  |  |  |  |  |
| Country | South Africa | 83 (17) | 29 (30) | 112 (19) | <.001** |
|  | Malawi | 83 (17) | 47 (48) | 130 (23) |  |
|  | Zambia | 0 (0) | 1 (1) | 1 (<0.5) |  |
|  | Uganda | 136 (28) | 6 (6) | 142 (25) |  |
|  | Zimbabwe | 176 (37) | 13 (13) | 189 (33) |  |
|  | Tanzania | 0 (0) | 1 (1) | 1 (<0.5) |  |
|  |  |  |  |  |  |
| AP Period | Period 1 | 80 (17) | 15 (15) | 95 (17) | 0.76** |
|  | Period 2 | 398 (83) | 82 (85) | 480 (83) |  |
|  |  |  |  |  |  |
| Weight (kg) | N | 478 | 97 | 575 | 0.45* |
|  | Median (Q1, Q3) | 65.0 (58.7, 75.0) | 64.5 (58.0, 72.1) | 64.7 (58.7, 74.5) |  |
|  |  |  |  |  |  |
| CD4 Cell Count (cells/mm^3^) | N | 478 | 97 | 575 | 0.84* |
|  | Median (Q1, Q3) | 544.0 (434.0, 684.0) | 536.0 (445.0, 670.0) | 544.0 (436.0, 684.0) |  |
|  |  |  |  |  |  |
|  | < 350 | 4 (1) | 0 (0) | 4 (1) | 0.53** |
|  | 350 - < 400 | 63 (13) | 12 (12) | 75 (13) |  |
|  | 400 - < 450 | 75 (16) | 14 (14) | 89 (15) |  |
|  | 450 - < 500 | 50 (10) | 16 (16) | 66 (11) |  |
|  | 500 - < 750 | 211 (44) | 38 (39) | 249 (43) |  |
|  | ≥ 750 | 75 (16) | 17 (18) | 92 (16) |  |
|  |  |  |  |  |  |
| HIV RNA level (copies/mL) | N | 478 | 97 | 575 | 0.45* |
|  | Median (Q1, Q3) | 8142.0 (1569.0, 31663.0) | 6403.0 (2121.0, 21131.0) | 7893.0 (1610.0, 30175.0) |  |
|  |  |  |  |  |  |
|  | Below lower limit of quantification (LLQ) of the assay | 24 (5) | 3 (3) | 27 (5) | 0.14** |
|  | <400 | 26 (5) | 11 (11) | 37 (6) |  |
|  | 400 - 1000 | 35 (7) | 3 (3) | 38 (7) |  |
|  | 1000- <10000 | 171 (36) | 39 (40) | 210 (37) |  |
|  | 10000 - <100000 | 175 (37) | 36 (37) | 211 (37) |  |
|  | 100000 - <200000 | 26 (5) | 3 (3) | 29 (5) |  |
|  | ≥200000 | 21 (4) | 2 (2) | 23 (4) |  |
|  |  |  |  |  |  |
| WHO Clinical Stage | Clinical stage I | 461 (96) | 93 (96) | 554 (96) | 0.77** |
|  | Clinical stage II | 17 (4) | 4 (4) | 21 (4) |  |
|  |  |  |  |  |  |
| HBsAg | Positive | 91 (19) | 19 (20) | 110 (19) | 0.90** |
|  | Negative | 387 (81) | 78 (80) | 465 (81) |  |
|  |  |  |  |  |  |
| Gestational age at Randomization (weeks) | N | 478 | 97 | 575 | 0.81* |
|  | Median (Q1, Q3) | 27.0 (21.9, 32.0) | 25.9 (21.1, 32.4) | 27.0 (21.7, 32.0) |  |
|  |  |  |  |  |  |
|  | < 14 | 3 (1) | 0 (0) | 3 (1) | 0.54** |
|  | 14 - < 28 | 259 (54) | 54 (56) | 313 (54) |  |
|  | 28 - < 34 | 137 (29) | 23 (24) | 160 (28) |  |
|  | ≥ 34 | 79 (17) | 20 (21) | 99 (17) |  |
|  |  |  |  |  |  |
| Calculated CrCl (mL/min) | N | 478 | 97 | 575 | 0.26* |
|  | Median (Q1, Q3) | 172.6 (146.3, 211.0) | 169.0 (139.8, 197.8) | 171.3 (144.5, 208.4) |  |
|  |  |  |  |  |  |
|  | >60 - 80 | 2/478 (<0.5) | 0/97 (0) | 2/575 (<0.5) | >0.99** |
|  | >80 - 100 | 4/478 (1) | 1/97 (1) | 5/575 (1) |  |
|  | >100 - 120 | 34/478 (7) | 6/97 (6) | 40/575 (7) |  |
|  | > 120 | 438/478 (92) | 90/97 (93) | 528/575 (92) |  |
| *Kruskal-Wallis Test **Chi-Square/ Fishers Exact Test  TDF = tenofovir disoproxil fumarate; ZDV = zidovudine; Q1 = 1^st^ Quartile; Q3 = 3^rd^ Quartile; AP = Antepartum; HBsAg = Hepatitis B Surface Antigen; CrCl = creatinine clearance; | | | | | |
