# Supplementary material for: Maternal and infant renal safety following tenofovir disoproxil fumarate exposure during pregnancy in a randomized control trial
Source: BMC Infect Dis. 2022 Jul 20;22:634. doi: 10.1186/s12879-022-07608-8 (PMC9297643; doi:10.1186/s12879-022-07608-8)
Supplement: Supplementary file 4 — Additional file 4: Table S1. PROMISE Study Follow-up Time and Tenofovir Disoproxil Fumarate (TDF) Exposure for Women Eligible for TDF Randomization. [file 12879_2022_7608_MOESM4_ESM.docx]

**Additional File 4 Table 1.** PROMISE Study Follow-up Time and Tenofovir Disoproxil Fumarate (TDF) Exposure for Women Eligible for TDF Randomization.

|  |  | **TDF-ART (N=445)** | **ZDV-ART (N=447)** | **ZDV Alone (N=446)** | **Total (N=1338)** |
| --- | --- | --- | --- | --- | --- |
| Follow-up at START Study Censor Date (Weeks) | N | 445 | 447 | 446 | 1338 |
|  | Median (Q1, Q3) | 80.0 (62.4, 100.0) | 80.4 (63.6, 101.0) | 79.2 (62.9, 99.7) | 79.9 (62.7, 100.4) |
|  |  |  |  |  |  |
| On Study at Start of Week 74 Window | Yes | 229 (51) | 234 (52) | 227 (51) | 690 (52) |
|  | No | 216 (49) | 213 (48) | 219 (49) | 648 (48) |
|  |  |  |  |  |  |
| Time on Regimen from Randomization to Delivery (Weeks) | N | 427 | 435 | 439 | 1301 |
|  | Median (Q1, Q3) | 11.7 (5.9, 15.9) | 11.4 (6.3, 16.6) | 12.1 (7.1, 17.0) | 11.9 (6.6, 16.6) |
|  |  |  |  |  |  |
| **Study Visit** | **On TDF-Containing Regimen*** |  |  |  |  |
| Delivery | No | 33/427 (8) | 426/434 (98) | 412/440 (94) | 871/1301 (67) |
|  | Yes | 394/427 (92) | 8/434 (2) | 28/440 (6) | 430/1301 (33) |
|  |  |  |  |  |  |
| Week 6 | No | 235/423 (56) | 219/431 (51) | 245/437 (56) | 699/1291 (54) |
|  | Yes | 188/423 (44) | 212/431 (49) | 192/437 (44) | 592/1291 (46) |
|  |  |  |  |  |  |
| Week 26 | No | 216/409 (53) | 208/419 (50) | 223/420 (53) | 647/1248 (52) |
|  | Yes | 193/409 (47) | 211/419 (50) | 197/420 (47) | 601/1248 (48) |
|  |  |  |  |  |  |
| Week 74 | No | 84/185 (45) | 103/178 (58) | 97/175 (55) | 284/538 (53) |
|  | Yes | 101/185 (55) | 75/178 (42) | 78/175 (45) | 254/538 (47) |
| *Excluding TDF tail  TDF = tenofovir disoproxil fumarate; ZDV = zidovudine; Q1 = 1^st^ Quartile; Q3 = 3^rd^ Quartile | | | | | |
