# Supplementary material for: Maternal and infant renal safety following tenofovir disoproxil fumarate exposure during pregnancy in a randomized control trial
Source: BMC Infect Dis. 2022 Jul 20;22:634. doi: 10.1186/s12879-022-07608-8 (PMC9297643; doi:10.1186/s12879-022-07608-8)
Supplement: Supplementary file 5 — Additional file 5: Supplementary and Sensitivity Analyses. [file 12879_2022_7608_MOESM5_ESM.docx]

**Methods**

*Supplementary Analyses*

Modification of the treatment effect by baseline subgroups (i.e., interaction tests) for the primary outcome measures were assessed using linear regression as a supplementary analysis. A two-sided P value of less than 0.05 was used to indicate a significant interaction. We assessed the interaction effects of the AP protocol version (Period 1 versus Period 2) and GA (< 34 weeks versus ≥ 34 weeks) at randomization. The analysis that stratified by GA at randomization was performed to assess the effect of differential TDF exposure times on the primary outcome.

Further supplementary analyses for the primary and select secondary and additional outcome measures used linear regression models to adjust for baseline covariates, including included: plasma HIV RNA, HBsAg status, GA, and CD4 cell count for women, and GA at birth for infants.

*Sensitivity Analyses*

All primary analyses applied a complete case approach that assumed data were missing completely at random (MCAR). To assess the assumptions of this approach, we used multiple imputation (MI) as a *post-hoc* sensitivity analysis for the primary outcome measures. We conducted the MI under the assumptions of missing at random (MAR), which implies that conditional on observed values, missingness of any variable does not depend on the unobserved values (i.e., the probability that a value is missing depends only on the observed data and not the value itself). We used a fully conditional specified (FCS) model with 30 burn-in iterations to create 1,000 imputed data sets. The imputation model included: randomization arm, AP Period, country, age at randomization, baseline BMI, HBsAg status at screening, baseline CrCl, GA at randomization, baseline CD4 cell count, and baseline RNA viral load. Analyses were completed as described in the primary analysis and results were combined using Rubin’s rules.

We also used the Wilcoxon rank test to compare the primary outcome measure across arms as a *post-hoc* non-parametric sensitivity analysis.

**Results**

*Supplementary Analyses*

Additional File 5 Table 1 summarizes all supplementary and sensitivity analyses. For women randomized during Period 1, the mean difference in calculated CrCl at Delivery between the TDF-Based ART regimen and the ZDV-Based ART regimen was -3.2 mL/min (-26.7, 20.3), whereas for women randomized during Period 2, the difference was -8.4 mL/min (-15.1, -1.7). The treatment effect did not differ significantly by enrollment Period (P = 0.68). Of note, there were only 31 women in the TDF-Based ART arm during Period 1, and per design eligibility, these women were HBsAg+. Differences in mean calculated CrCl at Delivery were also not significantly different for women randomized at an early GA (< 34 weeks) compared with women randomized at a later GA (≥ 34 weeks) (P = 0.94).

For infants born to women enrolled in the AP exposure part of the P1084s substudy, the mean difference in calculated CrCl at Birth between the TDF-Based ART arm and the ZDV-Based ART arm was -3.0 mL/min per 1.73 m^2^ (-17.2, 11.1) for infants born to women enrolled in the AP exposure part of the P1084s substudy during Period 1. This difference was -2.0 mL/min per 1.73 m^2^ (-8.3, 4.3) for between for infants born to women enrolled in the AP exposure part of the P1084s substudy during Period 2 (P = 0.89). Differences in mean calculated CrCl at Birth were also not significantly different for infants born to women randomized at an early GA(< 34 weeks) compared with later GA (≥ 34 weeks) (P = 0.63).

Between arm differences were adjusted for HBsAg status, CD4 cell count, log transformed HIV RNA level, and GA at randomization. The mean differences between the TDF-Based ART arm and the ZDV-Based ART arm remained significant after adjustment (P = 0.031), and treatment effect estimates did not change substantially (<8% change from unadjusted treatment effect estimate). For infants born to women enrolled in the AP exposure part of the P1084s substudy, there were no significant differences in mean calculated CrCl at Birth between arms after adjusting for GA at birth (P = 0.5), and the treatment effect estimate did not change substantially (20% change from unadjusted treatment effect estimate).

*Sensitivity Analyses*

Approximately 5% of expected values of calculated CrCl at Delivery was missing for all AP component women. Overall conclusions did not change following the imputation of missing values under the assumption of MAR. For infants born to women enrolled in the AP exposure part of the P1084s substudy who survived through 14 days of life, approximately 24% of calculated CrCl data at Birth were missing. Overall conclusions did not change following the imputation of missing values under the assumption of MAR.

**Additional File 5 Table 1.** Summary of Supplementary and Sensitivity Analyses.

| **Analysis Set** | **Outcome Measure** | **Analysis** | **Statistic** | **TDF-ART - ZDV-ART** | **P Value** |
| --- | --- | --- | --- | --- | --- |
| Women eligible for TDF randomization | Calculated CrCl (mL/min) | Unadjusted | Mean difference (95% CI) | -8.0 (-14.5, -1.5) | 0.014 |
|  |  | Protocol Version: Period 1 | Mean difference (95% CI) | -3.2 (-26.7, 20.3) | 0.68* |
|  |  | Protocol Version: Period 2 | Mean difference (95% CI) | -8.4 (-15.1, -1.7) |  |
|  |  | GA: <34 Weeks | Mean difference (95% CI) | -7.8 (-14.8, -0.9) | 0.94* |
|  |  | GA: ≥34 Weeks | Mean difference (95% CI) | -7.2 (-24.4, 10.1) |  |
|  |  | Adjusted for HBsAg status, CD4 cell count, log10 HIV RNA, and GA at randomization | Mean difference (95% CI) | -7.4 (-13.8, -1.0) | 0.031 |
|  |  | Multiple Imputation | Mean difference (95% CI) | -8.0 (-14.4, -1.6) | 0.014 |
|  |  | Wilcoxon | TDF-ART Median (Q1, Q3) | 141 (115, 171) | 0.001 |
|  |  |  | ZDV-ART Median (Q1, Q3) | 149 (125, 176) |  |
|  |  |  |  |  |  |
| Infants born to women enrolled in the AP exposure part of the P1084s substudy | Calculated CrCl (mL/min per 1.73m^2^) | Unadjusted | Mean difference (95% CI) | -2.5 (-8.4, 3.5) | 0.42 |
|  |  | Protocol Version: Period 1 | Mean difference (95% CI) | -3.0 (-17.2, 11.1) | 0.89* |
|  |  | Protocol Version: Period 2 | Mean difference (95% CI) | -2.0 (-8.3, 4.3) |  |
|  |  | GA: <34 Weeks | Mean difference (95% CI) | -5.8 (-22.4, 10.8) | 0.63* |
|  |  | GA: ≥34 Weeks | Mean difference (95% CI) | -1.4 (-7.7, 4.8) |  |
|  |  | Adjusted for GA at birth | Mean difference (95% CI) | -2.0 (-7.9, 3.9) | 0.50 |
|  |  | Multiple Imputation | Mean difference (95% CI) | -1.6 (-7.5, 4.2) | 0.58 |
|  |  | Including value below lower limit of quantification** | Mean difference (95% CI) | 13.2 (-18.3, 44.7) | 0.41 |
|  |  | Wilcoxon | TDF-ART Median (Q1, Q3) | 56.9 (44.1, 68.8) | 0.77 |
|  |  |  | ZDV-ART Median (Q1, Q3) | 57.4 (45.2, 71.3) |  |
| *P value is for the interaction term  **Serum creatinine value below lower limit of quantification was imputed as 0.009 mg/dL  TDF = tenofovir disoproxil fumarate; ZDV = zidovudine; ART = Antiretroviral Therapy; CrCl = Creatinine Clearance; CI = Confidence Interval; Q1 = 25^th^ Percentile; Q3 = 75^th^ Percentile | | | | | |
